# Machine Learning-Based Fault Location for Smart Distribution Networks Equipped with Micro-PMU

**DOI:** 10.3390/s22030945

**Published:** 2022-01-26

**Authors:** Hamid Mirshekali, Rahman Dashti, Ahmad Keshavarz, Hamid Reza Shaker

**Affiliations:** 1 Clinical-Laboratory Center of Power System & Protection, Faculty of Intelligent Systems Engineering and Data Science, Persian Gulf University, Bushehr 7516913817, Iran; h.mirshekali@mehr.pgu.ac.ir; 2 IoT and Signal Processing Research Group, ICT Research Institute Engineering Department, Faculty of Intelligent Systems Engineering and Data Science, Persian Gulf University, Bushehr 7516913817, Iran; 3 Center for Energy Informatics, The Maersk Mc-Kinney Moller Institute, University of Southern Denmark, 5230 Odense, Denmark

**Keywords:** machine learning, support vector machine, fault section location, micro-phasor measurement units, neighborhood component analysis

## Abstract

Faults in distribution networks occur unpredictably, causing a threat to public safety and resulting in power outages. Automated, efficient, and precise detection of faulty sections could be a major element in immediately restoring networks and avoiding further financial losses. Distributed generations (DGs) are used in smart distribution networks and have varied current levels and internal impedances. However, fault characteristics are completely unknown because of their stochastic nature. Therefore, in these circumstances, locating the fault might be difficult. However, as technology advances, micro-phasor measurement units (micro-PMU) are becoming more extensively employed in smart distribution networks, and might be a useful tool for reducing protection uncertainties. In this paper, a new machine learning-based fault location method is proposed for use regardless of fault characteristics and DG performance using recorded data of micro-PMUs during a fault. This method only uses the recorded voltage at the sub-station and DGs. The frequency component of the voltage signals is selected as a feature vector. The neighborhood component feature selection (NCFS) algorithm is utilized to extract more informative features and lower the feature vector dimension. A support vector machine (SVM) classifier is then applied to the decreased dimension training data. The simulations of various fault types are performed on the 11-node IEEE standard feeder equipped with three DGs. Results reveal that the accuracy of the proposed fault section identification algorithm is notable.

## 1. Introduction

Transient and hazardous situations can arise in distribution networks due to various types of uncertainties such as loading circumstances, distributed generations (DGs) performances, and defective scenarios [1]. Some may be identified and repaired easily, while others, such as line faults, can be caused by an external object or equipment failure. Faults in distribution network lines are unavoidable, and they cannot be prevented easily [2]. Furthermore, the inaccurate identification of a fault point may delay the network’s recovery time, leading to greater financial losses and customer discontent [3]. Therefore, in order to improve network dependability, faulty areas must be diagnosed quickly and accurately. With the advancement of technology, PMUs can be utilized in electricity networks to enable automatic fault location [4]. A micro-PMU is a type of PMU that is designed to record the quantities of the medium voltage side of a distribution network [5]. Micro-PMUs can record the voltage and current of the nodes and branches of the network synchronously. These recorded data can be used for monitoring, control, design, and diagnostic purposes so that operators can comprehend the state of the distribution network in real time [6]. Using these devices in the distribution networks makes automatic data-driven fault location possible. There has been a wide range of approaches for locating distribution network faults. Impedance-based [7], traveling wave-based [8], and intelligent approaches [9] are among the most widely deployed fault location methods. A comprehensive review of different types of fault location methods and their own sets of advantages and disadvantages are reported in [10]. However, in the following, a brief review of several related works is given.

Impedance-based fault location methods mostly apply only sub-station voltage and current to determine the location of the fault in the distribution network. Using phase domain equations and low-resolution data of fault, are two main advantages of this method [11]. In [12], a new fault location method is presented that uses smart meter data to determine faulty points more accurately. The main benefit of this work is using low-resolution data for determining possible fault locations. However, it needs higher resolution data to determine the real location of the fault in multi-branch networks. This method can be applied on fully and non-fully observable networks and requires both recorded voltage and current at the sub-station and DGs. Using current waveform can compromise the fault location procedure for short circuit faults with high current amplitude because of the CT saturation. An additional difficulty of applying fault current information could be the summation of measurement error [13]. A smart feeder meter is a measuring device that is installed in a medium voltage node of a distribution network and has the task of measuring the voltage of that node and the current of each connected branch [14]. Since impedance-based methods exhibit several responses for fault spots in an extended distribution network, in [15], a new method is presented to distinguish the faulty section of the network. The smart meters are located in the branches with more than two sections to support every section of the network. The pre- and post-fault active power of each branch are calculated using recorded voltage and current. For a faulty section, the injected current to the fault point has a considerable value compared to the rest of the points. One of the main drawbacks of this work is its dependency on smart meter data and loading conditions. DGs are one of the main components of smart grids. However, they increase the complexity of the network, which results in a more complex fault location procedure [16]. In [17], a new algorithm is presented to locate faults in non-looped distribution networks equipped with DGs. In this work, a new impedance-based approach is employed to identify the faulty section. One of the main disadvantages of this work is the need for exact load values of all nodes requiring special measuring devices, which is not cost-effective.

Travelling wave-based approaches are among the most popular methods for finding the location of faults in distribution networks. The main advantage of these methods is their accuracy and their ability to function independently from the network condition. However, they need high sampling rate devices to work effectively in low-distance distribution networks. Using these devices in the distribution network is not recommended from a financial perspective [18]. In traveling wave-based methods, the arrival time of the voltage and current signals from the fault spot is essential to solve fault location equations related to the traveling signals. The precision of the arrival time is highly dependent on the decomposition and scale level of the signal, which is related to the sampling frequency [19]. In [20], a new method is presented to address this high sampling frequency problem. A new image-to-image translation method is presented that uses a scale one detail component image acquired by low sampling rate measurements. The main drawback of this work is that it needs two measurements from both sides of a section to determine the location of fault, which is not practical in real-world networks. Distribution networks have low-distance feeders and sections with a considerable number of branches because of their urban nature. Therefore, the main challenge could be finding a faulty section rather than the fault distance in that section. In [21], a new wide-area measurement-based method is presented to determine the faulty section of a complex distribution network by employing traveling wave data acquisition. The arrival time of traveling waves from pre-determined measurements (located in a specific manner in the network) are obtained using traveling wavelet and phase-mode transformation. The accurate measuring integration is determined utilizing Manhattan distance for the initial faulty spot. Then, the accurate location of the fault is calculated with the monitoring room information, which is network topology and fault occurrence time for each measuring device. One of this work’s drawbacks is the need for accurate fault time that requires synchronized measurements. The secondary disadvantage of this paper is its use of high sampling rate devices, which is impractical for real-world networks.

Machine learning (ML) strategies are one of the most promising techniques for identifying faults in the smart distribution networks due to their adaptability and effectiveness [22]. A convolution wavelet extreme learning machine has been used to develop a coherent platform for fault identification and localization [23]. Feature extraction is incorporated into the process of learning in this approach. The fact that no line parameters are needed is a key benefit of this approach. In the first step of the procedure, the type of fault is established, and then the location is determined. The main drawbacks of this method are its complexity and its need for the current signal for functionality. Smart feeder meters can be widely used in distribution networks and serve as a useful tool for fault location. A gated recurrent unit block is a simplified model of a long short-term memory unit that eliminates some complex mathematical functions [24]. In [25], a new framework based on deep learning that employs a special type of recurrent neural network called the gated recurrent unit to locate faulty sections in the network is suggested. In this study, smart feeder meters are assumed to be installed in all network nodes, which is its primary disadvantage due to their high cost. The main benefit of this work is its use of only two samples of different angles before and after the fault, which is ultra-low resolution. Further, its dependency on the fault type could be indicated. This implies that regardless of the kind of fault or its characteristics, all recorded data from all measures are inputted into the algorithm in real time. SVM classifiers are one of the most popular classifiers in machine learning frameworks. In [26], a new hybrid impedance SVM-based fault location method is presented for distribution networks. In the first step, the recorded data of voltage and current at the sub-station are fed to the impedance-based algorithm to locate all possible distances of the fault. Then, an online data bank is generated using the simulated data of possible fault location with the fault data and the available information of the network in the monitoring center. An SVM is trained to map each set of data to its own class or possible faulty sections. The sub-stations’ recorded data are then sent into the classifier, which determines which class it belongs to. In this method, it is crucial to have both recorded voltage and current data of faults, which is a downside of this strategy. In addition, in this research, the effect of the DGs in the transient condition of the network is not considered. Compressed sensing is a signal processing approach that seeks solutions to inherently unknowable linear algebra to effectively acquire and recreate a signal. This is predicated on the idea that by optimizing a signal’s sparsity, it may be recovered with significantly fewer samples than the Nyquist–Shannon sampling theorem requires. This technique can be used for the sparse-measurement-based fault location methods to estimate the position of faults in the distribution network with or without DGs [27]. In [28], a new compressive sensing-based method is suggested to locate faults in distribution networks equipped with smart feeder meters. In this work, the smart feeder meters record three-phase voltage sags of different nodes of the network. Then, the network’s faulty node can be determined using the compressed sensing method and $l^{1}$-norm minimization. The main advantage of this work is that it does not use the loading condition of consumers. The main drawback of this work is its inability to perform with only the resource’s recorded data. In [29], the same procedure as in [28] is selected for fault localization. In this work, both the faulty node and the faulty branch can be detected. Table 1 compares the characteristics of several methods to those of the proposed method, which simplifies the understanding of these works.

In this paper, a new machine learning-based fault location is presented for smart distribution networks equipped with DGs. Micro-PMU recorded voltage data of fault at the sub-station and DGs are utilized. The sampling rate is set to 5 kHz, which provides the first 100 harmonics of the signal. In Clark transformation, the alpha component of the ABC sequenced voltage signal is acquired. It transfers three vectors of data to one vector, which makes the learning process much simpler and faster. Frequency spectrum analysis is then used to extract the harmonics of the voltage waveform as the feature vectors. Because of the problem’s complexity and to prevent unnecessary computation, neighborhood component analysis (NCA) is then applied to the feature vectors. NCFS algorithm employs training labeled data set to remove low-value features from the feature vectors for increasing classification accuracy. The SVM classification method is then applied to the training data set. The linear kernel is selected for the sake of simplicity and computational burden. Different types of fault scenarios are simulated to evaluate the power of the proposed method. The simulation environment is MATLAB Simulink 2020b. An IEEE 11-node test feeder equipped with three DGs of different types is considered. Four micro-PMUs are located in this network in nodes 1, 9, 10, and 11. A data set of the fault with various characteristics of resistances and locations are generated. The main contributions of this research are as follows:

- A novel non-iterative fault location method is presented to identify the faulty sections in the smart distribution network equipped with only DGs using voltage data.
- This strategy uses only the recorded voltage waveform at the sub-station as well as any other network resources with a sampling rate of 5 kHz.
- The proposed machine learning-based method is not sensitive to fault characteristics and functions in real-time without any extra information of protection relays.

The rest of the paper is organized as follows. In Section 2, a new method to identify the faulty section is described in three subsections. The simulation results and performance evaluation are demonstrated in Section 3. In the last section, the conclusion is reached. 

## 2. Proposed Method

In this paper, the recorded voltage waveform at the sub-station and all DGs were utilized for fault section identification purposes. The proposed method is described in the following three parts.

### 2.1. Data Set

One quarter and three quarters pre- and post- fault voltage data of a cycle from all micro-PMUs will be gathered and fed to the proposed fault location algorithm. In the first step of the method, all of the simulated data of different types of faults in every location of the network each with its own characteristics must be used to train an SVM classifier. For simplicity and to cover every type of fault, the Clark transform will be used [34]. The alpha component of the voltage signal will be used. The transformation will be done as follows:

(1) $$\left[\begin{array}{c} v_{\alpha }\\ v_{\beta }\\ v_{0} \end{array}\right]=\left[\begin{array}{ccc} \frac{2}{3} & -\frac{1}{3} & -\frac{1}{3}\\ 0 & \frac{1}{\sqrt{3}} & -\frac{1}{\sqrt{3}}\\ \frac{1}{3} & \frac{1}{3} & \frac{1}{3} \end{array}\right]\left[\begin{array}{c} v_{a}\\ v_{b}\\ v_{c} \end{array}\right]$$

**Figure 1:**
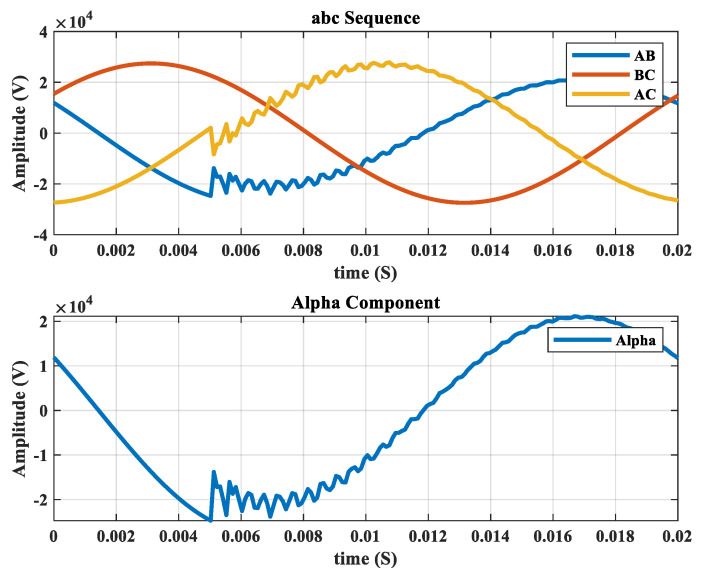
ABC sequence and alpha component of voltage waveform for a sample fault.

**Figure 2:**
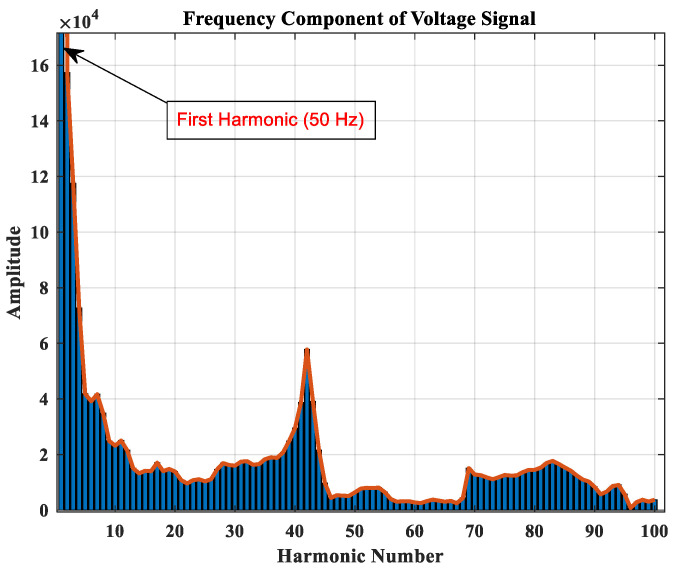
Frequency component of a sample fault in the network.

Figure 1 shows the ABC sequence and alpha component of a faulty signal of voltage in the same frame. After Clark transformation, another step to achieving more informative data from the sinusoidal signal of fault will be required. For this purpose, frequency component analysis of the recorded voltages of all resources in the grid could be an excellent option. Fast Fourier transform (FFT) of the voltages could be used. The sampling frequency is considered to be 5000 Hz, which provides the first 100 harmonics. These harmonics are selected as the features of each fault case. For instance, consider the IEEE 11-node test feeder with three DGs in nodes 9, 10, and 11. The recorded voltage waveform at the sub-station is plotted in Figure 1 for a 10-ohm single phase to ground fault. The frequency component of this experiment is extracted and is shown in Figure 2. There will be four vectors of 100 features (sub-station and three DGs) for each fault, which complicates the training process. Therefore, in the next section, the NCA method is used to extract more informative features from each vector.

### 2.2. Neighborhood Component Analysis

One step of the machine learning procedure that has a significant impact on its performance is feature extraction. There are several methods that can be used for finding more informative features from the input data to the algorithm. NCA is a non-parametric strategy that selects features using the nearest neighbor decision rule with the purpose of improving the prediction accuracy of regression and classification algorithms. When compared to state-of-the-art classification approaches such as SVMs and neural networks, the nearest neighbor is a simple and efficient non-linear decision process that often produces competitive results. In this part, the NCFS algorithm proposed in [35] is briefly reviewed. NCFS applies on the faulty data for identifying more effective features to decrease the dimension of the feature vector. Lowering the dimension of the feature vector by eliminating the less valuable features can enhance the accuracy of the classifier. With a regularization term, this approach employs gradient ascent to optimize expected leave-one-out classification accuracy. Assume the following data set:

(2) $$S=\left\{(X_{1},y_{11}),\;\left(X_{2},y_{21}\right),\;\ldots \left(X_{i},y_{ij}\right),\;\ldots \left(X_{N},y_{NJ}\right)\right\}$$

where S is the class of training data set that contains N labeled samples. $X_{i}$ is the i-th d-dimensional feature vector and $y_{ij}$ is its corresponding class with j={1, …,J} representing the class number. The weighted distance function between each sample is defined as follows:

(3) $$D_{\Omega }\left(X_{i},X_{z}\right)=\overset{d}{\underset{m=1}{\sum }}\omega_{m}^{2}\left|x_{im}-x_{zm}\right|$$

where $D_{\Omega }$ is the weighting distance function of two samples, d is the number of features, and $\Omega =\left\{\omega_{1},\;\ldots \;,\;\omega_{d}\right\}$ is a d-dimensional weighting vector that represents the value of each corresponding feature in the classification procedure. The goal of this function is to find the optimal weighting vector Ω, which determines the most effective features based on the nearest neighbor. To achieve this, leave-one-out classification accuracy must be maximized on the given training data set S. Since, in this method, each sample needs a true reference point to perform properly, a function is defined that calculates the probability of $X_{i}$ choosing $X_{z}$ as its reference point. The maximum probability for each sample determines its reference point. The probability function is defined as follows:

(4) $$P_{iz}=\{\begin{array}{ll} \frac{k\left(D_{\Omega }\left(X_{i},X_{z}\right)\right)}{\sum_{k\neq i}k\left(D_{\Omega }\left(X_{i},X_{z}\right)\right)}, & i\neq j\\ 0, & i=j \end{array}$$

In this equation, k(r)=exp(r/σ) is a kernel function with kernel width σ. The value of σ determines the chance of each point being selected as the reference point. The probability of a point $X_{i}$ being correctly classified is as follows:

(5) $$P_{i}=\underset{z}{\sum }E_{iz}P_{iz}$$

where $E_{iz}=1$ if $y_{ij}=y_{iz}$, otherwise $E_{iz}=0$. Hence, leave-one-out classification accuracy approximation can be described as follows:

(6) $$\zeta \left(\Omega \right)=\underset{i}{\sum }\underset{z}{\sum }y_{iz}p_{iz}-\lambda \overset{d}{\underset{m=1}{\sum }}\omega_{m}^{2}$$

where λ>0 is a regularization term that can be adjusted with cross-validation. The derivative of the objective function with respect to $\omega_{m}$ is as follows:

(7) $$\frac{\partial \zeta \left(\Omega \right)}{\partial \omega_{m}}=2\left(\frac{1}{\sigma }\sum_{i}\left(p_{i}\sum_{z\neq i}P_{iz}\left|x_{im}-x_{zm}\right|-\sum_{z}y_{iz}P_{iz}\left|x_{im}-x_{zm}\right|\right)-\lambda \right)\omega_{m}$$

The above formula leads to a similar gradient ascent iterative equation. The optimal value of Ω can be computed iteratively for Equations (5)–(7). Since the weighting vector Ω indicates each feature’s value, those features with larger values than a threshold can be determined to be the main features and used in the SVM classifier. The SVM classifier is described briefly in the next part.

### 2.3. Support Vector Machine Classifier

**Figure 3:**
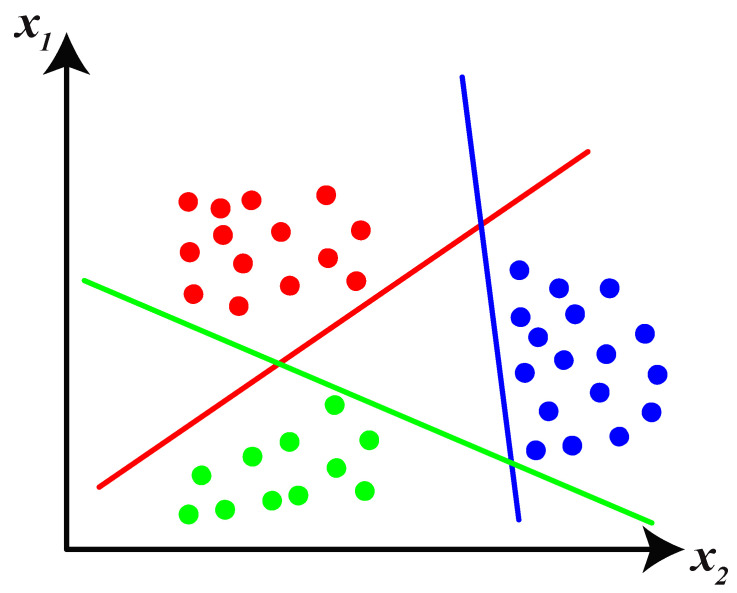
SVM classifier representation with linear kernel for two-dimensional data set.

SVM is one of the most popular machine learning methods that uses supervised learning and models to classify or regress a discrete or continuous set of data, respectively. An SVM learning algorithm creates a model that allocates training examples to one or another specified category, which results in generating a non-probabilistic binary linear classifier. However, there are some methods, such as Platt scaling, that utilize SVM in a probabilistic manner. SVM projects training data vectors to locate in space in order to expand the distance between any categories as much as possible. The test data vectors are then projected into the same specified area and classified according to which part of the space they stand on [36]. Figure 3 shows the functionality of SVM in a two-dimensional space. There are three classes that are divided by three lines. SVM has several advantages that make it a suitable choice for the proposed faulty section identification problem. In the suggested method, the recorded voltage of DGs and the sub-station is used to train the SVM model. For the networks with a considerable number of DGs, the features exceed the amount of training data. SVM is a suitable tool for the training procedure in this scenario because it is more effective for high-dimensional data. On the other hand, SVM is relatively more memory efficient than KNN, which results in a lower computational burden than KNN. In this paper, an SVM classifier with a linear kernel is used to classify each fault data. The paper’s aim is to find the maximum margin hyperplanes for each label using training data so that the distance between the hyperplanes and the closest data of each class is maximized. SVM classifies the data set with linear boundaries. For higher dimensional data vectors, the hyperplanes must be used to segment each area in the space. The output of the proposed feature selection algorithm is data vectors with more than 10 features. Because the feature vectors are multi-dimensional (each fault type has more than 10 characteristics), a set of hyperplanes should be used to determine which class a data vector belongs to. In the case of two classes and a data set of $x_{i}$ training data and $y_{i}$ target classes of 1 and −1, the following formulas are used:

(8) $$\{\begin{array}{ll} x_{i}\in \omega_{1} & if\;y_{i}=1\\ x_{i}\in \omega_{2} & if\;y_{i}=-1 \end{array}$$

where ω is a vector of the following classification interface, which can be trained by the given training data set.

(9) $$y_{i}\left(\omega^{T}x_{i}+b\right)\geq 1,\;\;\;\;\;\;\;i=1,\;\ldots ,\;N.$$

**Figure 4:**
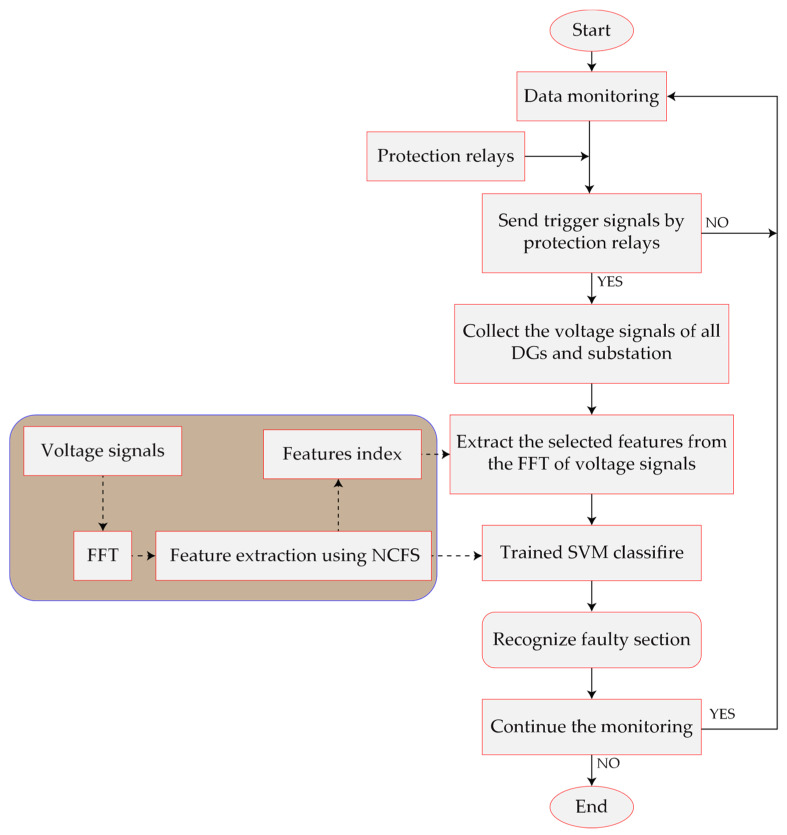
The flowchart of the proposed method.

The sampling point distance to the classification interface that contains the weighting vector ω must be maximized to achieve a sufficient classifier. In order to achieve this, ‖ω‖ must be minimized under the determined constraints of each class’ hyperplanes. The final kernel classification function is written as follows:

(10) $$d\left(x\right)=sgn\left(\overset{n}{\underset{i=1}{\sum }}a_{i}^{*}y_{i}K\left(x_{i},x\right)+b^{*}\right)$$

where $a_{i}^{*}$ and $b^{*}$ are weighting coefficient and classification threshold. $K\left(x_{i},x\right)$ is a kernel function that could be linear or non–linear. The flowchart of the proposed method is shown in Figure 4. According to this flowchart, a new platform for identifying faulty sections is presented using simulation data of a fault. The training process is performed in a way that this platform could identify the faulty section with higher accuracy, which results in a centralized intelligence. After a fault occurs in the network, the protection relay sends a command of fault to the pre-trained platform. The platform collects the data of all micro-PMUs in the network for the faulty spot identification process. Therefore, a communication network is necessary to gather the recorded data of micro-PMUs. In this method, there is no need for communication between micro-PMUs. The distribution networks usually have a low distance between their sections, which results in a low distance between DGs and the monitoring center. Therefore, it is possible to send the recorded data to the monitoring center for more analysis through an Internet of Things (IoT) platform. In order to execute the fault location algorithm, the recorded data must be sent to the monitoring center by means of sensors and wireless modules in the IoT platform. The estimated time to send information depends on the type of communication equipment that is used in the network. However, the execution time of the fault location algorithm is less than 100 milliseconds. Several factors must be considered in order for the proposed method to be used in real-world networks. The first is that special devices, such as micro-PMUs, are required to record the voltage of several pre-determined nodes in the network (nodes with resources). The second step is to communicate the captured data to the monitoring center through wireless modules. The third object is a communication link that is sufficiently secure against potential cyber-attacks. Furthermore, for simulation and data generation, the whole topology of the network, including all alternative topologies, must be provided. In summary, to practically apply this strategy in a real-world distribution network, metering devices, wireless data transmission modules, and IoT-based resistance (against cyber-attacks) communication platforms are required. The proposed method can only detect the faulty section and cannot predict it. As a result, if a fault occurs in a section of a smart distribution network equipped with DGs, the network might still deliver power to all consumers by removing the faulty section from the network using automated breakers on both sides of that section. The entire operation, from detecting the faulty section to isolating it, could be automated. The only difficulty would be locating the faulty section, which the suggested fault location approach would address. Algorithm 1 shows both the offline training process and the real-time fault location process. In this paper, a linear kernel is used for simplicity and computational burden. In the next part, the results are presented and discussed.
**Algorithm 1. Machine Learning-Based Fault Location****Input—pre-trained platform, recorded voltage of micro-PMUs****Offline process**: Training process1:Simulate the real-world feeder using monitoring room information for different fault scenarios 2:Gather the recorded voltage data of all fault scenarios3:Extract the alpha component of the voltage signals 4:Perform frequency spectrum analysis of the voltage signals and generate feature vectors5:Extract more informative features of training data vectors to lower the dimension of feature vectors using NCFS algorithm6:Attach each feature vector label to prepare for the training process7:Train the SVM to determine the linear boundary of each class with hyperplanes8:The machine learning-based fault location platform is ready 9:End**Real-time process**: Fault location1:Monitor the network2:If the protection relay sends the trigger signals, then collect the recorded voltage data, otherwise go back to Step 13:Perform frequency spectrum analysis of the voltage signals4:Extract the pre-determined features using the feature extraction index 5:Feed the data to the pre-trained SVM6:Print the determined class as the faulty section7:Monitor the network as in Step 18:End 




## 3. Simulation Results

**Figure 5:**
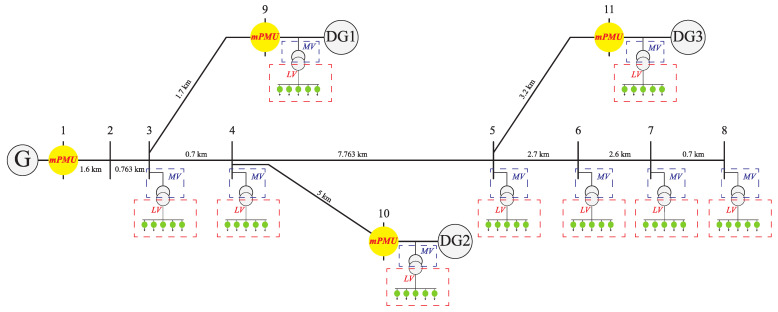
IEEE 11-node test feeder equipped with DGs.

To evaluate the effectiveness of the proposed machine learning-based fault location strategy simulations were performed. For this purpose, a standard 11-node IEEE test system equipped with DGs was selected, which is depicted in Figure 5. This network contained 11 nodes, 10 sections, and 3 DGs, which were located in nodes 9, 10, and 11 of the network. DGs located in nodes 9, 10, and 11 have 4, 8, and 6 MVA power, respectively, with X/R = 5. Although distribution networks have a short distance between each node and usually their sections were modeled, to take the capacitive nature of the lines into account and gather a more precise training data set, the pi line model was utilized. The simulation environment was Simulink MATLAB 2020b.

**Figure 6:**
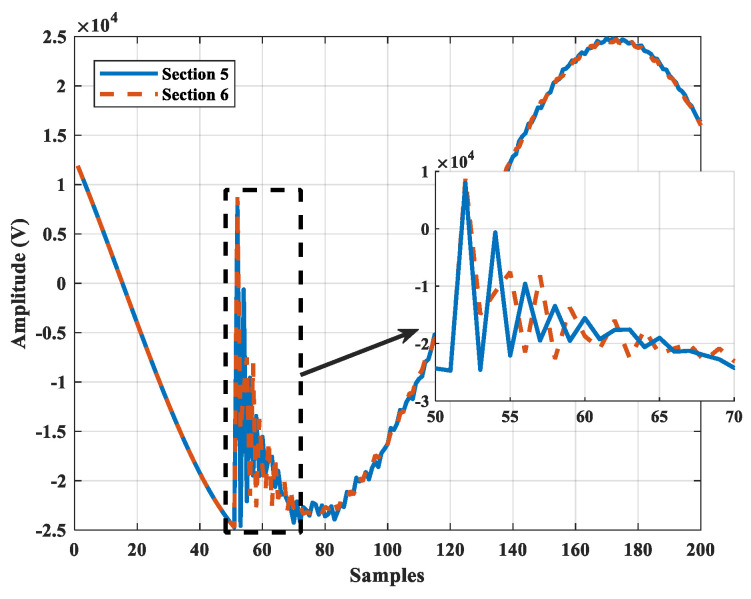
Alpha component of two sample faults in Sections 5 and 6.

**Figure 7:**
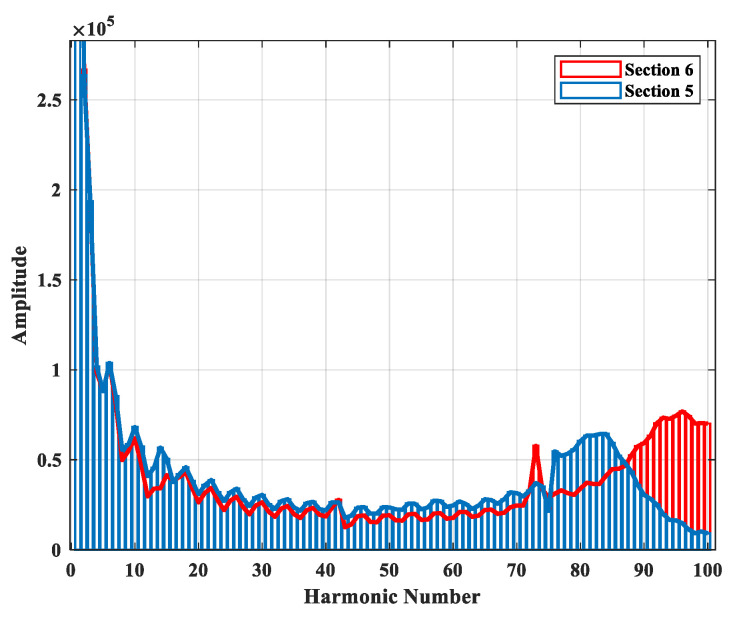
Frequency spectrum of two sample faults in Sections 5 and 6.

**Figure 8:**
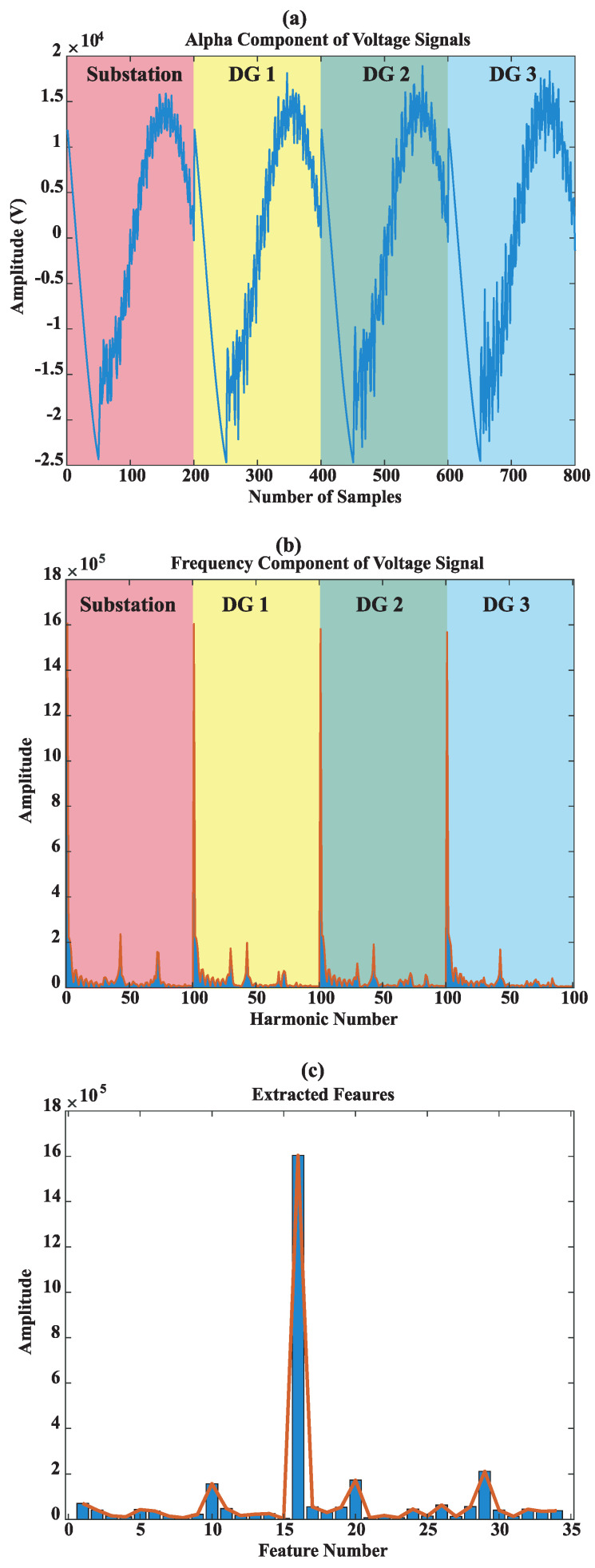
A sample fault, (**a**) alpha component voltage of all resources and the sub-station, (**b**) the frequency spectrum of voltage signals, (**c**) extracted the more informative features from the rest features.

Figure 6 and Figure 7 depict the alpha component and frequency spectrum of two single-phase-to-ground faults in Sections 5 and 6. As can be seen the same fault in the different sections have different higher-order harmonics. Note that only sub-station voltage is pictured. All scenarios of fault are given in Table 2, including a total of 990 for each case of fault type. For single-phase-to-ground fault of 1-ohm resistance, the voltage waveforms of the sub-station and all DGs are depicted in Figure 8a. The first 100 harmonics of the four voltage signals are shown in Figure 8b. The NCFS algorithm was applied to the training data set and 35 harmonics out of 400 harmonics were extracted to determine the most effective for the classification procedure. Figure 8c presents the selected harmonics with the highest efficiency. 

**Figure 9:**
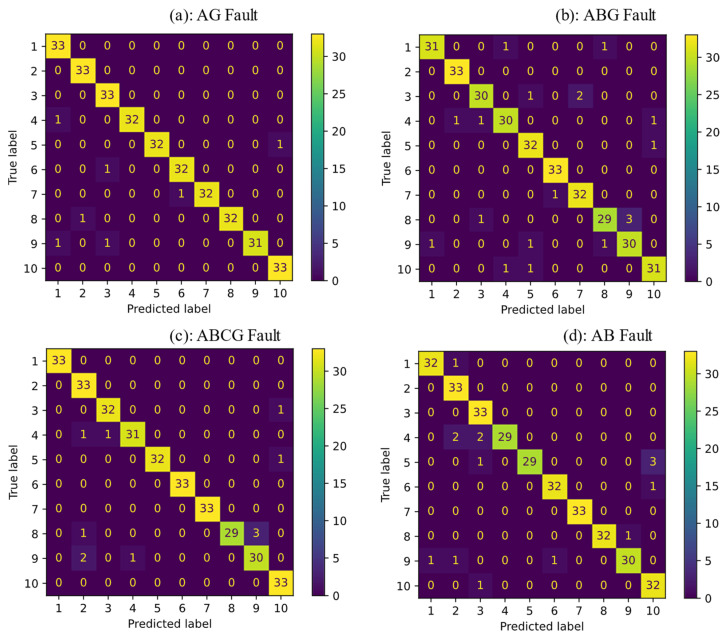
The confusion matrix for four types of fault scenarios, (**a**) Single-phase to ground fault, (**b**) Two-phase to ground fault, (**c**) Three-phase to ground fault, (**d**) Phase-to-phase fault.

**Figure 10:**
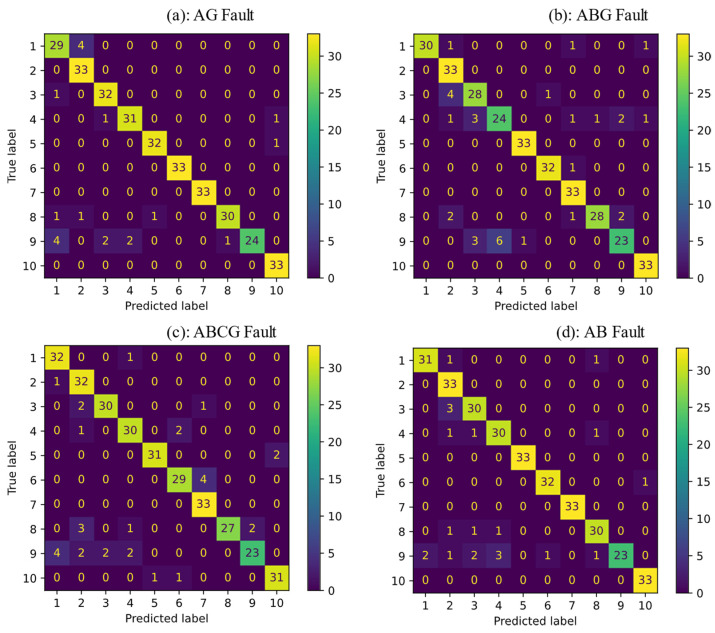
The confusion matrix for four types of faults using KNN, (**a**) Single-phase to ground fault, (**b**) Two-phase to ground fault, (**c**) Three-phase to ground fault, (**d**) Phase-to-phase fault.

The SVM classifier was trained for single-phase-to-ground (AG), two-phase-to-ground (ABG), three-phase-to-ground (ABCG), and phase-to-phase (AB) faults. Each type of fault was simulated with different fault resistances of 1, 5, 10, 15, 20, 25, 30, 35, 40, 45, and 50 ohms. There were 10 sections in the test network, which resulted in 10 classes. The data set was divided into 70% train and 30% test data set. 30% data of each class was considered for the testing process. The accuracy percentage of the classifier for AG, ABG, ABCG, and AB ere 97.87%, 94.24%, 96.66%, and 95.45%, respectively. A confusion matrix, often referred to as an error matrix, is a table structure that enables analysis of the effectiveness of a technique, almost always a supervised learning one, used in the machine learning field and particularly in the study of statistical classification. A confusion matrix is a table that shows the performance of the proposed classifier in detail. It has d×d dimensions that d is the numbers of the target. The vertical label represents true classes and the horizontal one shows the predicted classes with the proposed classifier. The confusion matrix demonstrates how many classes were predicted correctly. It also shows which classes could not be properly detected. The main diameter shows the number or percentage of true detected classes. It demonstrates how much of the training data of a class was detected correctly. Figure 9 shows the confusion matrixes for four fault types. As can be seen, 7 classes of AG fault, 19 classes of ABG fault, 11 classes of ABCG fault, and 15 classes of AB fault could not be detected correctly out of 330 total faults. This number of faulty section misdetection is acceptable for distribution networks with short sections lines and uncertain DGs while using only voltage signals. The proposed method was accurate in terms of faulty section detection. However, for the sake of comparison, the method of [37] was also simulated and the results were presented. This work used the K-nearest neighbor (KNN) algorithm to determine the location of a fault in the electrical grid. The generated simulation data of fault were fed to the algorithm. Note that for a fair comparison, frequency spectrum analysis was performed on the data. Then, the same features of the proposed method’s data were selected to create the input vectors to the KNN algorithm. The comparative results of the accuracy of both methods are reported in Table 3. For several cases of single-, two-, and three-phase-to-ground fault and phase-to-phase fault, a classifier was trained. The main assumption was that the same training data that was generated and passed through the pre-processing block is used for the classification process. Since the distribution of the training data was more linear, SVM could classify more accurately than KNN. The identical training and test data sets were fed to SVM and KNN classifiers in the scenario of single-phase fault, resulting in 97.87% and 93.93% accuracy, respectively. The proposed method accuracy was higher than the KNN algorithm for all cases of fault. In addition, the confusion matrix is also plotted in Figure 10. As can be seen from the two confusion matrices, SVM has fewer false negative and false positive predictions compared to the KNN algorithm, which highlights the benefits of the proposed method.

## 4. Conclusions

Faults may happen in electrical networks. The ability to detect faulty sections in smart distribution networks equipped with DGs accurately and in real time is critical for meeting customer demand and avoiding long-term shutdowns and financial losses. In the paper, a new machine learning-based fault section detection was introduced. This work used the recorded voltage at the sub-station and DGs with the low sampling rate of 5 kHz, which were measured by micro-PMU. One cycle of data with a quarter before fault and three-quarters after fault was considered as a data window for each sample. Frequency spectrum analysis of the voltage signals was used to extract the required features. Then, the NCFS algorithm was applied to the training data set to distinguish more informative features from the rest within a threshold. Then, the new training data set was fed to an SVM classifier with a linear kernel. The simulations of different fault scenarios were performed on the 11-node IEEE test feeder in a MATLAB 2020b environment. The training data were used for both the SVM and KNN methods. Based on the results, it was concluded that the proposed approach is accurate despite lack of access to current signals and other nodes’ recorded information.

The proposed method can improve network reliability while also being cost effective by restoring the network quickly and precisely. The initial investment might include the communication link, metering equipment, and wireless sensors. If a failure happens in a traditional network without this equipment, it would take several hours to locate the faulty area and repair the damaged section, resulting in financial losses and customer dissatisfaction. The financial loss may vary depending on the kind of consumer (domestic, commercial, or industrial). The cost of an outage for a large company may easily reach millions of dollars per hour of downtime. Because the network could be restored quickly and safely using this approach, the financial damage could be reduced. The return on investment is determined by the network type, the number and location of faults in the network, the fault location platform equipment utilized, and the customer type.

## Figures and Tables

**Table 1:** The comparison of the methods.

Characteristics/References	[30]	[31]	[32]	[33]	Proposed Method
Network type	R/L	R/L	R/L	R	R/L
Line type	DLM	DLM	DLM	DLM	DLM
Method type	CNN	NBC–SVM–ELM	GCN	ANN	SVM
Features	Wavelet	HHT	Phasor	Wavelet	FFT
Data type	Voltage and current	Current	Voltage and current	Current	Voltage
Fault type	All	All	All	All	All
DG	Yes	Yes	No	No	Yes
Feature extraction	Automatic	No	No	No	NCA
Complexity	High	Low	Normal	Normal	Low
Number of measurements	All nodes	All nodes	Limited nodes	At the sub-station	Equal to the resources
Advantages	1, 2, 3, 14,	2, 3, 7, 12, 14, 23	1, 2, 9, 14, 22	2, 3, 19	2, 3, 7, 12, 14, 15, 20, 23
Disadvantages	4, 5, 6, 11, 16, 18	4, 13, 17, 18	4, 6, 9, 10, 11, 13, 16, 21	4, 5, 6, 8, 11, 17	4, 13

DLM: distributed line model, CNN: convolutional neural network, NBC: naive base classifier, ELM: extreme learning machine, HHT: Hilbert–Huang transform, GCN: graph convolutional network, ANN: artificial neural network, R: radial, L: loop, 1: There is no need to know fault type, 2: Does not need the load value, 3: Does not need line parameters, 4: Needs data bank, 5: Needs high sampling rate devices, 6: Complex structure, 7: Simple structure, 8: Inapplicable for loop network, 9: High inaccuracy in face of unbalanced load, 10: Impractical against DG high penetration, 11: High computational burden, 12: Estimating faulty section, 13: Cannot identify the exact location of fault, 14: Applicable for loop grids, 15: Only needs voltage signal, 16: Needs both current and voltage signal, 17: Can operate with only current signal, 18: Needs measuring devices in all nodes, 19: Measurements only at the sub-station, 20: Only needs the recorded data of resources, 21: Needs limited number of measurements, 22: Uses phase domain equations, 23: Low computational burden.

**Table 2:** The simulation parameters details.

Parameters	Details	Count
Structure	Radial	1
Line sections	All lines of 11-node ieee bus	10
Fault type	AG, ABG, ABCG, AB	4
Fault spots in each section	10%, 20%, 30%, 40%, 50% 60%, 70%, 80% and 90% of each section	9
Fault resistance	1, 5, 10, 15, 20, 25, 30, 35, 40, 45, and 50 Ω	11
All scenarios	Each fault type	990

**Table 3:** The accuracy of SVM and KNN against different fault types.

Methods\Fault Type	AG	ABG	ABCG	AB
SVM	97.87%	94.24%	96.66%	95.45%
KNN	93.93%	90%	90.3%	93.33%

## Data Availability

Not Applicable.
